# Soluble urokinase plasminogen activator receptor is associated with short-term mortality and enhanced reactive oxygen species production in acute-on-chronic liver failure

**DOI:** 10.1186/s12876-021-02006-x

**Published:** 2021-11-17

**Authors:** Yunyun Wang, Fengtian Wu, Chao Chen, Lichen Xu, Wei Lin, Chunhong Huang, Ying Yang, Shanshan Wu, Jinjin Qi, Hanqin Cao, Guojun Li, Meng Hong, Haihong Zhu

**Affiliations:** 1 State Key Laboratory for Diagnosis and Treatment of Infectious Diseases, Collaborative Innovation Center for Diagnosis and Treatment of Infectious Disease, The First Affiliated Hospital, School of Medicine, Zhejiang University, 79 Qingchun Road, Shangcheng District, Hangzhou, 310003 Zhejiang China; 2 Infectious Diseases Department, The First Affiliated Hospital of Wenzhou Medical University, Wenzhou, Zhejiang China; 3 The Third Affiliated Hospital of Wenzhou Medical University, Wenzhou, Zhejiang China; 4 Clinical Laboratory, The First Affiliated Hospital, School of Medicine, Zhejiang University, Hangzhou, Zhejiang China; 5 Hepatology Department, Ningbo Yinzhou No. 2 Hospital, Ningbo, Zhejiang China

**Keywords:** suPAR, HBV, ACLF, Mortality, ROS

## Abstract

**Background:**

Acute-on-chronic liver failure (ACLF) is a comprehensive syndrome characterized by an acute deterioration of liver function and high short-term mortality rates in patients with chronic liver disease. Whether plasma soluble urokinase plasminogen activator receptor (suPAR) is a suitable biomarker for the prognosis of patients with ACLF remains unknown.

**Method:**

A prospective cohort of 282 patients with ACLF from three hospitals in China was included. 88.4% of the group was hepatitis B virus-related ACLF (HBV-related ACLF). Cox regression was used to assess the impact of plasma suPAR and other factors on 30- and 90-day mortality. Reactive oxygen species (ROS) production were detected to explore the role of suPAR in regulating neutrophil function in HBV-related ACLF.

**Result:**

There was no difference in plasma suPAR levels between HBV-related and non-HBV-related ACLF. Patients with clinical complications had higher suPAR levels than those without these complications. A significant correlation was also found between suPAR and prognostic scores, infection indicators and inflammatory cytokines. Cox’s regression multivariate analysis identified suPAR ≥ 14.7 ng/mL as a predictor for both day 30 and 90 mortality (Area under the ROC curve: 0.751 and 0.742 respectively), independent of the MELD and SOFA scores in patients with ACLF. Moreover, we firstly discovered suPAR enhanced neutrophil ROS production under *E.coli* stimulation in patients with HBV-related ACLF.

**Conclusions:**

suPAR was a useful independent biomarker of short-term outcomes in patients with ACLF and might play a key role in the pathogenesis.

*Trial registration* CNT, NCT02965560.

**Supplementary Information:**

The online version contains supplementary material available at 10.1186/s12876-021-02006-x.

## Background

Acute-on-chronic liver failure (ACLF) is a complex syndrome defined by the acute onset of liver failure in patients with pre-existing chronic liver disease. It is characterized by high short-term mortality, organ failure, and overwhelming systemic inflammation [1, 2]. Excessive systemic inflammations are believed to be the key driver for the development of ACLF [2].

Model for End-Stage Liver Disease (MELD), MELD-Na and Child-Turcotte-Pugh (CTP) scores, the conventional scoring systems, do not accurately predict mortality and multi-organ failure (MOF) in ACLF. Recently the CANONIC study developed the CLIF-consortium organ failure (CLIF-C) score, which was demonstrated to be more useful for predicting the outcome of ACLF than conventional scoring systems [2]. However, the scoring process is a little complicated which might impede the understanding of patient conditions in time. Thus, new biomarkers with good predictive value are needed to be discovered [3–5].

Urokinase-type plasminogen activator receptor (uPAR) is a part of the plasminogen activator (PA) system. This system is involved in many physiological and pathological processes, including thrombosis [6], inflammation [7], tissue remodeling [8] and tumourigenesis [9]. soluble urokinase plasminogen activator receptor (suPAR) is a stable protein, released from cleavage of urokinase plasminogen activator receptor (uPAR, CD87) during inflammation [10–12]. uPAR is mainly expressed on the membranes of circulating immune cells such as monocytes and neutrophils and is closely associated with immune functions such as cell attachment, motility, migration, proliferation, and fibrinolysis [12–14]. suPAR retains most of activities of uPAR [10, 15]. Moreover, suPAR could bind to podocyte β3 integrin to cause kidney disease [16] and potentiate lipopolysaccharide-induced neutrophil activation [17].

Reactive oxygen species (ROS) plays a key role in acute liver injury. It recruited inflammatory cells to liver site, killed normal cells, resulted in mitochondrial dysfunction and promotes the secretion of cytokines [18, 19].

The role of suPAR on regulating reactive oxygen species (ROS) production remains unknown. Levels of suPAR are elevated in various infections like HIV infection, malaria, tuberculosis, and sepsis, suggesting its potential ability to predict the outcome of these diseases [20]. This predictive ability might also be useful in ACLF, but few studies have focused on the level of suPAR in patients with ACLF. Moreover, the measurement of suPAR is simple and fast (done in 1 h 40 min) by a commercial ELISA kit. Thus, we explored whether suPAR was also an appropriate biomarker for determining prognosis in ACLF and its role on regulating ROS productions in neutrophils.

## Methods

### Patients

**Fig. 1:**
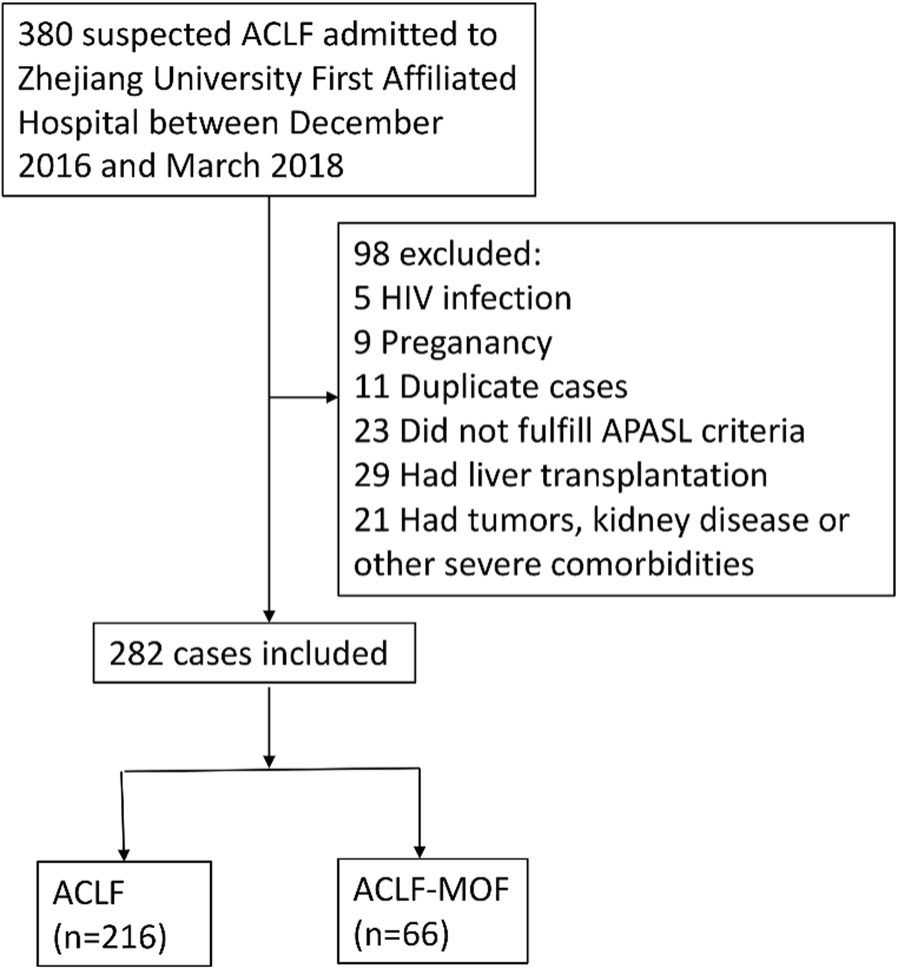
Flow diagram of patient selection. ACLF-MOF indicated the patients with ACLF developed MOF during 90-day follow-up

In this prospective study, adult patients suspected to have ACLF and admitted to the Zhejiang University First Affiliated Hospital (Hangzhou, China), Provincial Youth People's Hospital (Hangzhou, China) and Ningbo Yinzhou No.2 Hospital (Ningbo, China) between December 10, 2016 and March 10, 2018 were recruited (Fig. 1). Since the patients included were all from China, the diagnosis of ACLF was based on the Asian-Pacific Association for the Study of the Liver (APASL) criteria: “acute hepatic insult manifesting as jaundice (bilirubin ≥ 5 mg/dl) and coagulopathy (INR > 1.5) complicated within 4 weeks by ascites and/or encephalopathy in a patient with previously diagnosed or undiagnosed chronic liver disease” [21]. Cirrhosis was diagnosed by previous liver biopsy, endoscopy, radiological evidence, or clinical manifestation of liver decompensation. Hepatorenal syndrome (HRS), spontaneous bacterial peritonitis (SBP), and ascites were diagnosed using the criteria established by the International Ascites Club and American Association for the Study of Liver Disease, respectively [22, 23]. Patients with ACLF were then further classified as acute-on-chronic liver failure with multi-organ failure (ACLF-MOF) based on the presence of two or more extra-hepatic organ failures and others as ACLF [24]. Patients were excluded if they were pregnant, diagnosed with acquired immunodeficiency syndrome (AIDS), had any type of malignant tumor, or had undergone liver transplantation. Chronic hepatitis B (CHB) was defined as patients with stable chronic hepatitis B, which was diagnosed by histology or imaging or laboratorial or clinical evidence of cirrhosis or liver fibrosis or long-term liver inflammation together with serum HBsAg positive for more than six months. Healthy controls (HC) had no history or clinical evidence of previous or present illness and with serum HBsAg negative. CHB and HC were gender and age matched with ACLF patients. This study met the principles of the Helsinki Declaration and was approved by the ethics committee of Zhejiang University First Affiliated Hospital. Written consent was acquired from each participant or their legal representative. The study cohort was followed for 90 days after enrolment and the end point was set as either death or liver transplantation.

### Sample size calculation

Sample size was calculated by using logrank tests (hazard rate) on PASS software. Tests power was set at 1−β = 0.9, significance level was set at α = 0.05 (two sided). Follow up lost rate was considered as 15%.

### suPAR and cytokines measurement

Whole blood samples from participants were collected within two days after study enrollment. After centrifugation, plasma was obtained and stored immediately at − 80 °C. Plasma samples (25μL) were used to measure suPAR using an enzyme-linked immunosorbent assay (ViroGates, Denmark), and 20μL of plasma sample was used to measure cytokines using a multiplex panel (Bio-Rad, Hercules, CA), according to the manufacturer’s instructions. The detection limits were in the supplementary methods (Additional file 1).

### Oxidative burst assays

100μL of whole blood samples from HBV-related ACLF, patients with chronic hepatitis B (CHB) or healthy controls (HC) were pre-incubated with suPAR(50 ng/mL, R&D, USA) or PBS for 45 min at 37 °C in 5% CO_2_. Next, all samples were incubated with heat-inactivated *E. coli* (8 × 10^7^ cfu/mL) in 96-well plates for 30 min. Then the cells were harvested for CD16-percp-cy5.5 (Biolegend, USA) staining and oxidative burst assessment using an ROS assay kit (Genecopoeia, MD, USA) and were analyzed by a LSRFortessa cytometer (BD bioscience, USA) according to the manufacturer’s instructions. Neutrophils were indicated as CD16^+^.

### Statistical analysis

Descriptive statistics were expressed as counts [%] and mean ± standard deviation (SD). Continuous data with a non-normal distribution were shown as median (interquartile range; IQR). Baseline characteristics were compared between patients with ACLF and ACLF-MOF by using the Mann–Whitney *U* test for continuous variables or Fischer’s exact/Pearson’s χ^2^ test for categorical variables. Spearman’s rank correlation analysis was conducted to investigate associations between plasma suPAR concentration and laboratory and clinical data. Due to the unavailability of data in some patients, this correlation analysis was not performed on all patients. Cox univariate and a further multivariate analysis were conducted to distinguish variables highly correlated with mortality. Odds ratios (ORs) and 95% confidence intervals (CIs) were determined for each variable. The ability to predict mortality was calculated by receiver operating characteristic curve (ROC curve) and the comparison of ROC curves was performed by MedCalc using DeLong's test. Kaplan Meier survival curves were also developed. Loss of follow-up would also be included in the survival analysis. Comparisons between paired groups were analyzed by the Wilcoxon signed-rank test. All statistical tests were two-sided and *p* < 0.05 was considered statistically significant.

## Results

### Patient characteristics

The estimated smallest sample size was 94. After screening, 282 patients with ACLF who fulfilled the inclusion and exclusion criteria were recruited into the study (Fig. 1). The comparison of baseline characteristics of these patients with or without MOF is shown in Table 1. suPAR and baseline characteristics was measured in all participants. Plasma suPAR at admission was significantly higher in patients with MOF than those without MOF (11.9 (9.1–15.5) vs. 16.4 (11.5–24.0) ng/mL; *p* < 0.001, Table 1). Significant differences between the two groups were also found for the presence of some clinical events such as HRS and hepatic encephalopathy (HE), laboratory data such as white blood cell count (WBC), international normalized ratio (INR) and total bilirubin (Tbil), and prognostic scoring systems such as CTP, MELD and SOFA scores. All patients were followed at the end of the point.

**Table 1:** Baseline characteristics of ACLF patients

	(A)ACLF (n = 216)	(B)ACLF-MOF (n = 66)	(C)CHB (n = 14)	(D)HC (n = 14)	*p*- value A vs B
Age (years), Mean (± SD)	47.1 ± 12.6	46.4 ± 13.5	43.1 ± 14.3	42 ± 10.1	0.69
Male (%)	185 (85.6)	57(86.4)	12(85.7%)	12(85.7%)	0.88
Etiology					0.07
HBV (%)	191 (88.4)	64 (97)	14 (100)	–	
Others (%)	25 (11.6)	2 (3)	–	–	
Clinical feature					
Ascites (%)	170 (78.7)	50 (75.8)	–	–	0.61
Cirrhosis (%)	110 (50.9)	31 (47.0)	–	–	0.57
UGIB (%)	18 (8.3)	8 (12.1)	–	–	0.35
HRS (%)	5 (2.3)	9 (13.6)	–	–	< 0.001
HE (%)	7 (3.2)	24 (36.4)	–	–	< 0.001
SBP (%)	9 (4.2)	6 (9.1)	–	–	0.12
Bacterial or fungal infection (%)	23 (10.6)	12 (18.2)	–	–	0.10
Sepsis (%)	1 (0.5)	2 (3.0)	–	–	0.27
Laboratory data					
suPAR (ng/mL), Median (IQR)	11.9 (9.06–15.5)	16.4 (11.4–23.6)	2.8 (2.2–4.0)	2.3 (2.0–2.9)	< 0.001
WBC (× 10^9^/L), Median (IQR)	6.2 (4.8–8.7)	8.1 (6.1–12.6)	5.1 (4.3–6.1)	5.4 (4.2–6.8)	< 0.001
Platelets (× 10^9^/L), Median (IQR)	101 (72–140)	122 (81–177)	150 (116–200)	160 (110–231)	0.05
ALT (IU/L), Median (IQR)	190 (67–492)	267 (175–910)	45 (16–101)	18 (10–24)	0.001
Albumin (g/L), Mean (± SD)	31.1 ± 4.3	31.7 ± 4.0	40.5 ± 3.5	42.7 ± 3.0	0.39
Bilirubin (mg/dL), Median (IQR)	18.1 (12.7–24.3)	21.0 (15.2–26.6)	0.8 (0.5–1.0)	0.7 (0.4–0.9)	0.039
INR, Median (IQR)	1.94 (1.73–2.30)	3.14 (2.7–3.7)	–	–	< 0.001
Creatinine (mg/dL), Median (IQR)	0.7 (0.6–0.9)	0.8 (0.7–1.1)	0.6 (0.5–0.7)	0.6 (0.5–0.7)	0.04
Sodium (mmol/L), Mean (± SD)	137.2 ± 3.9	137.6 ± 4.8	140 ± 3.1	142 ± 2.0	0.56
Scores					
CTP, Mean (± SD)	10.8 ± 1.3	11.4 ± 1.7	–	–	0.007
MELD, Mean (± SD)	21.9 ± 4.6	30.4 ± 6.5	–	–	< 0.001
SOFA, Mean (± SD)	8.6 ± 1.76	12.0 ± 1.4	–	–	< 0.001

*ACLF* acute-on-chronic liver failure; *ACLF-MOF* ACLF complicated with multi-organ failure; *CHB* chronic hepatitis B; *HC* healthy controls; *UGIB* upper gastrointestinal bleeding; *HRS* hepatorenal syndrome; *HE* hepatic encephalopathy; *SBP* spontaneous bacterial peritonitis; *WBC* white blood cell count; *INR* international normalized ratio; *CTP* Child—Turcotte-Pugh; *MELD* Model for End-stage Liver Disease; *SOFA* sequential organ failure assessment

### Baseline plasma suPAR levels and association with ACLF disease progression

**Fig. 2:**
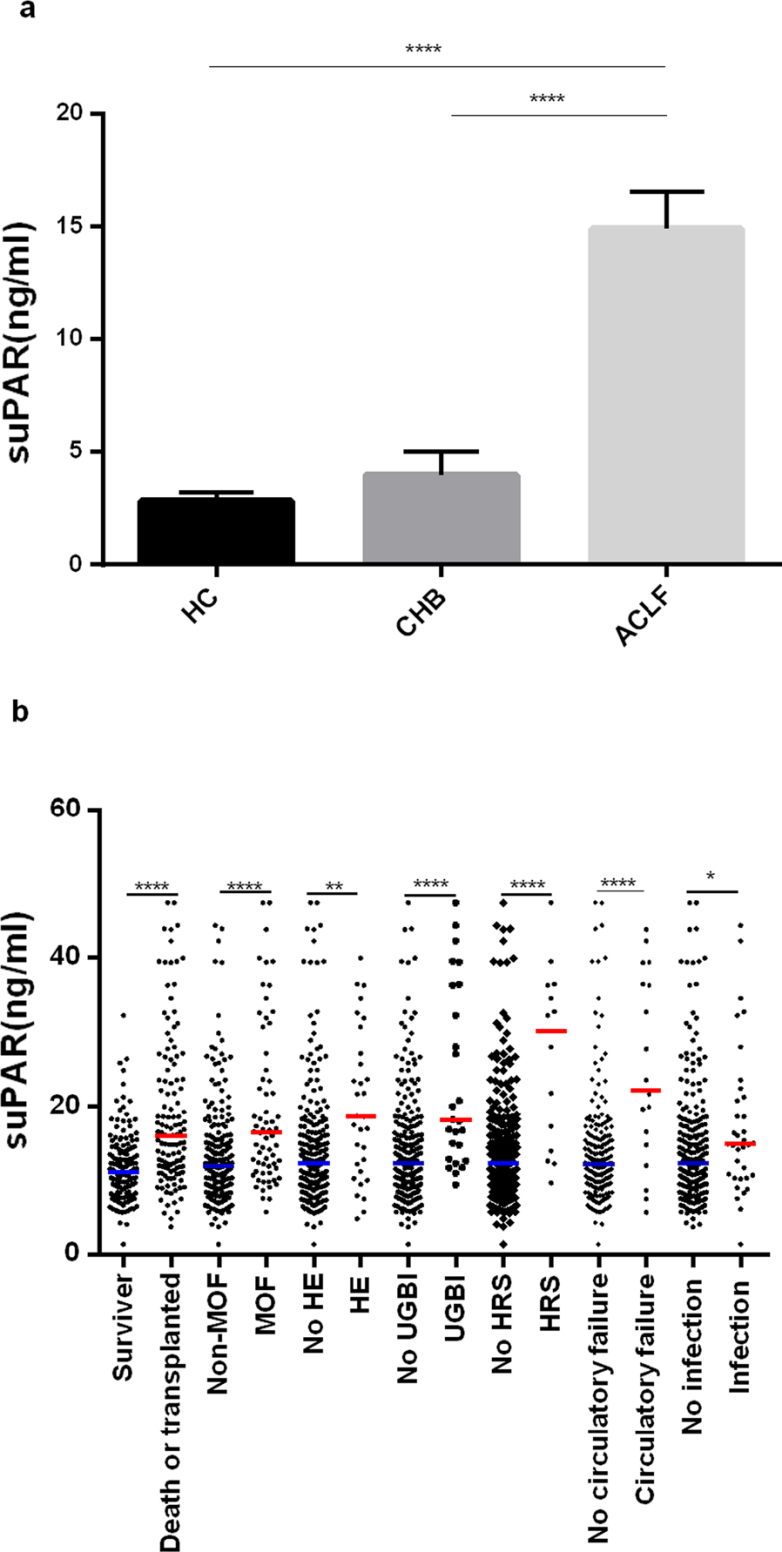
Comparison of plasma suPAR levels in different disease groups. **a** Distribution of plasma suPAR concentrations among HC (n = 14), CHB (n = 14) and ACLF (n = 42). **b** The comparison of suPAR levels between ACLF patients with and without clinical complications. Horizontal lines represent median values. *ns* not statistically significant; *p < 0.05; **p < 0.01, ***p < 0.001, ****p < 0.0001

There was no difference in plasma suPAR levels between HBV-related and non-HBV-related ACLF (*p* > 0.05, Additional file 1: Fig. A. S 1). We then determined plasma suPAR among HC, CHB and ACLF and found out suPAR levels in patients with ACLF were markedly higher than those with HC and CHB. (Fig. 2a, 12.16 (7.61–17.57) vs. 2.3 (2.00–2.89) vs 2.7 (2.16–4.00) ng/L; *p* < 0.001). However, no difference was shown between HC and CHB. We then further compared plasma suPAR levels in ACLF patients with or without complications at admission or during the follow-up period. At admission, patients with HE, HRS, UGBI or infection had higher suPAR levels than those without these complications. Differences in plasma suPAR levels were most pronounced in patients with HRS (30.15 (13.57–36.35) vs. 12.30 (9.33–16.46) ng/L; *p* < 0.001) (Fig. 2b). There was no statistically significant difference in suPAR levels between patients with or without SBP, ascites, cirrhosis (Additional file 1: Fig. A. S1). Patients exhibiting circulatory failure during follow-up also showed significantly higher suPAR levels than patients without circulatory failure (Fig. 2b).

Aside from clinical features, significant correlations with plasma suPAR were also found for clinical laboratory data and prognostic scores (Table 2). All three prognostic scores were correlated with suPAR levels; the strongest correlation was found with the MELD (*r* = 0.421, *p* < 0.001) (Table 2). Among the laboratory data, various infection-immunity related data showed a positive association with suPAR levels, including the incidence of bacterial or fungal infection, WBC and PCT. Interestingly, in white blood cells, suPAR was positively correlated with the percentage of neutrophils but did not correlate with the percentage of monocytes. At the same time, suPAR was also negatively correlated with HBcAb and the percentage of lymphocytes, indicating that suPAR was positively correlated with innate immunity, but negatively correlated with adaptive immunity in patients with HBV-related ACLF. As systemic inflammation seemed to be the driver for the development of organ failure in ACLF [2], we also investigated whether the concentration of plasma suPAR was associated with plasma inflammatory cytokines in patients with ACLF. After identifying 27 cytokines in 40 patients, a strong correlation was found between two chemokines, MIP1beta and IL-8, and plasma suPAR levels (*r* = 0.453, *p* = 0.007; *r* = 0.448, *p* = 0.003, respectively) (Table 2). Significant correlations were also found between suPAR and liver-related data, kidney-related data, and thyroid-related data, but not heart-related data (Table 2).

**Table 2:** Association of clinical parameters and prognostic scoring systems with serum suPAR concentrations

Variable	Correlation coefficient with serum suPAR (r)	*p* value	Number of patients
Age	0.203**	0.001	282
Infection-immunity data			
Bacterial or fungal infection	0.118*	0.04	282
HBcAb	− 0.182**	0.003	261
WBC	0.306***	< 0.001	282
Neutrophil (%)	0.250***	< 0.001	271
Monocyte (%)	0.065	0.28	271
Lymphocyte (%)	− 0.327***	< 0.001	271
PCT	0.205**	0.008	166
CPR	0.055	0.39	215
MIP1beta	0.453**	0.007	40
IL8	0.448**	0.003	40
Liver-related data			
ALT	0.036	0.55	282
Albumin	− 0.098	0.101	282
Bilirubin	0.287***	< 0.001	282
INR	0.281***	< 0.001	282
Heart-related data			
MAP	− 0.015	0.80	282
hsTnI	0.161	0.080	119
Kidney-related data			
GFR	− 0.290***	< 0.001	270
Creatinine	0.219***	< 0.001	282
Sodium	− 0.221***	< 0.001	282
Thyroid-related data			
T_3_	− 0.356***	< 0.001	254
FT_3_	− 0.270***	< 0.001	254
Scores			
CTP	0.183**	0.002	282
MELD	0.425***	< 0.001	282
SOFA	0.356***	< 0.001	282

*HBcAb* antibody against HBV core; *PCT* procalcitonin; *CRP* C-reactive protein; *MIP1beta* macrophage inflammatory protein 1-beta; *INR* international normalized ratio; *MAP* mean arterial pressure; *hsTnI* hypersensitive troponin I; *GFR* glomerular filtration rate; *T3* Total triiodothyronine; *FT3* free triiodothyronine; *CTP* Child—Turcotte-Pugh; *SOFA* sequential organ failure assessment, *MELD* Model for End-stage Liver Disease; *UGIB* upper gastrointestinal bleeding; *WBC* white blood cell count. *p < 0.05, **p < 0.01, ***p < 0.001

### Survival analysis

During the 30-day follow-up, sixty-two (22.0%) patients died and thirty-four (12.1%) received a liver transplant. During the 90-day follow-up, eighty-two (29.1%) patients died and forty-one (14.5%) underwent liver transplantation. The median follow-up time was 90 days (IQR: 26, 90).

Baseline plasma suPAR increased in patients who died or underwent transplant (n = 123) during the 90-cm day follow-up compared to those who survived without liver transplantation (n = 159) (16.03 (11.65–23.70) vs. 11.14 (8.41–14.14) ng/L, *p* < 0.001; Fig. 2b).

**Fig. 3:**
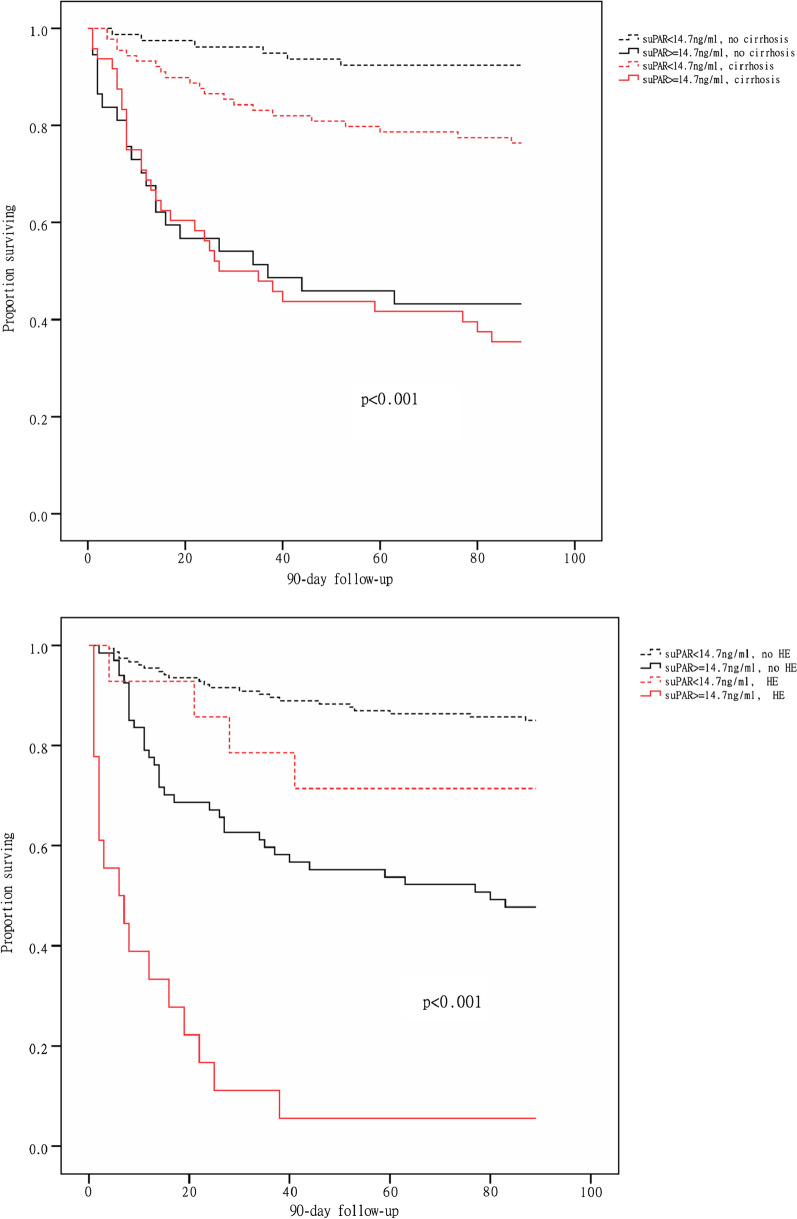
Comparison of K–M survival curves between ACLF patients with or without cirrhosis and with or without HE. The cumulative 90-day survival between groups was compared using the log-rank test

The optimal cut-off point for plasma suPAR in predicting 90-day mortality was 14.7 ng/mL, as calculated by the Youden Index. Based on this optimal cut-off point, Kaplan–Meier curves significantly indicated 30- and 90-day mortality for patients with ACLF. Intriguingly, the effect of suPAR levels on the mortality was significantly larger in patients without cirrhosis or with HE (Fig. 3). The 30-day mortality of patients with both ACLF and cirrhosis was not impacted as much as the 90-day mortality by high suPAR levels (Fig. 3). During 90-day follow-up, patients with high suPAR (suPAR ≥ 14.7 ng/mL) and with HE had the highest mortality while ACLF patients with low suPAR and without HE had the lowest mortality (Fig. 3).

Correlations between clinical features/laboratory data and short-term mortality were analyzed by univariate Cox regression (Additional file 1: Table A. S1). Results showed that suPAR was significantly associated with both 90-day and 30-day mortality (*p* < 0.001). Variables with statistically significant (*p* < 0.05) in univariate regression analyses and age were included in multivariate models. To explore whether serum suPAR was correlated with the short-term mortality independently of the prognostic scores, suPAR was separately evaluated with MELD and SOFA scores in multivariate analysis. Variables included in two prognostic scores would be ruled out from multivariate models in order avoid collinearity.

Cox’s regression multivariate analysis using the forward step-wise selection method identified suPAR ≥ 14.7 ng/mL and WBC ≥ 6.6 × 10^9^, together with MELD ≥ 23.1 SOFA ≥ 9.5, as the independent predictors of both day 90 and day 30 mortality (Table 3). These models were validated by bootstrapping.

**Table 3:** Multivariate Cox regression models for short-term mortality in ACLF patients

	HR (95% CI)	*p* value	Bootstrapping *p* value
Mortality at 30 days			
Model 1: MELD score			
MELD ≥ 23.1	3.62 (1.93–6.76)	< 0.001	0.001
suPAR ≥ 14.7	3.72 (2.15–6.41)	< 0.001	0.001
WBC ≥ 6.6	1.96 (1.10–3.51)	0.02	0.016
Model 2: SOFA score			
SOFA ≥ 9.5	3.18 (1.67–6.10)	< 0.001	0.001
suPAR ≥ 14.7	3.52 (2.02–6.13)	< 0.001	0.001
WBC ≥ 6.6	2.03 (1.13–3.67)	0.02	0.017
Model 3 Other Clinical Data			
suPAR ≥ 14.7	4.77 (2.78–8.19)	< 0.001	0.006
Sepsis	7.48 (2.25–24.85)	0.001	0.009
Cirrhosis	–	0.25	0.30
UGIB	–	0.40	0.78
HRS	–	0.09	0.15
Mortality at 90 days			
Model 1: MELD score			
MELD ≥ 23.1	3.19 (1.88–5.41)	< 0.001	0.001
suPAR ≥ 14.7	3.02 (1.90–4.81)	< 0.001	0.001
WBC ≥ 6.6	1.97 (1.21–3.21)	0.007	0.008
Age ≥ 46.5	2.51 (1.54–4.08)	< 0.001	0.003
Model 2: SOFA score			
SOFA ≥ 9.5	2.54 (1.51–4.29)	< 0.001	0.001
suPAR ≥ 14.7	2.89 (1.78–4.69)	< 0.001	0.001
WBC ≥ 6.6	2.00 (1.22–3.27)	0.006	0.007
Age ≥ 46.5	2.73 (1.68–4.45)	< 0.001	0.001
Model 3 Other Clinical Data			
suPAR ≥ 14.7	4.52 (2.87–7.14)	< 0.001	0.003
Sepsis	7.33 (2.21–24.32)	0.001	0.02
Cirrhosis	–	0.10	0.15
UGIB	–	0.24	0.58
HRS	–	0.12	0.24

*UGIB* upper gastrointestinal bleeding; *HRS* hepatorenal syndrome, *WBC* white blood cell count; *MELD* Model for End-stage Liver Disease; *SOFA* sequential organ failure assessment

Moreover, analysis of the area under the receiver operating characteristic curve (ROC-AUC) revealed that suPAR may be a useful predictor for both 30- and 90-day mortality in ACLF patients (0.751 and 0.742, respectively) (Table 4). Combining MELD or SOFA score with suPAR improved the ROC-AUC of the scores for predicting 90-day mortality (*p* = 0.03 and *p* = 0.002 respectively, Table 4). At 30 days of follow-up, ROC-AUC of the SOFA score, but not MELD score, for predicting mortality significantly improved by combining with serum suPAR (*p* = 0.03 and *p* = 0.059, respectively; Table 4). The combining equations were illustrated in Additional file 1.

**Table 4:** ROC area of suPAR predicting mortality in ACLF patients

	ROC area (95% CI)	*p* value
Mortality at 30 days		
suPAR	0.751 (0.684–0.817)	
MELD	0.732 (0.658–0.807)	Reference
MELD + suPAR	0.773 (0.709–0.837)	0.059
SOFA	0.763 (0.698–0.828)	Reference
SOFA + suPAR	0.798 (0.736–0.86)	0.030
Mortality at 90 days		
suPAR	0.742 (0.680–0.805)	
MELD	0.729 (0.663–0.795)	Reference
MELD + suPAR	0.780 (0.722,0.837)	0.030
SOFA	0.726 (0.662–0.789)	Reference
SOFA + suPAR	0.785 (0.727, 0.843)	0.002

*MELD* Model for End-stage Liver Disease; *SOFA* sequential organ failure assessment

### suPAR enhanced neutrophil ROS production under *E.coli* stimulation

**Fig. 4:**
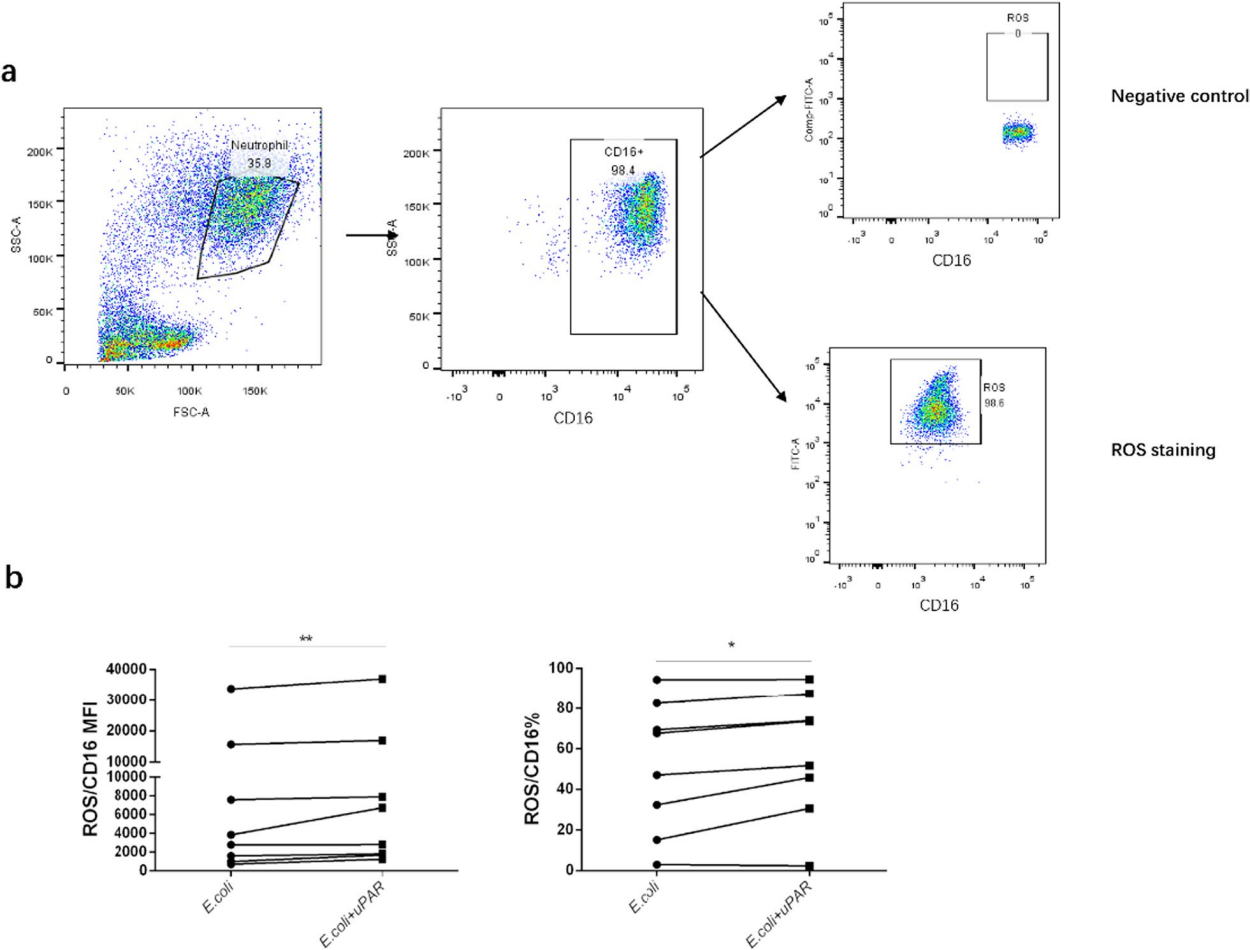
suPAR enhanced ROS production in neutrophils in HBV-related ACLF under *E.coli* stimulation. Whole blood from eight patients with HBV-related ACLF was stimulated with *E. coli* in the presence of suPAR (50 ng/ml) or PBS for 30 min in vitro. Gating Strategies for ROS detection of neutrophils was shown in (**a**). The impact of suPAR on neutrophil ROS production was determined in (**b**). Statistical analyses were performed using the Wilcoxon signed-rank test. *p < 0.05; **p < 0.01

ROS plays a key role in ACLF pathogenesis [25]. To better understand the role of suPAR in ACLF, we applied suPAR to circulating neutrophils from patients with HBV related-ACLF under stimulation of *E. coli.* There was enhanced ROS production in neutrophils after suPAR addition, suggesting elevated serum suPAR levels promote disease progress in HBV related-ACLF (Fig. 4, *p* = 0.008 and *p* = 0.023 respectively). As for patients with chronic hepatitis B (CHB), though the frequency of ROS^+^ neutrophils decreased quite slightly after the addition of suPAR (Additional file 1: Fig. A. S 2, *p* = 0.0425), the MFI of ROS did not change (Additional file 1: Fig. A. S 2, *p* = 0.771). And there was no significant effect of suPAR on ROS levels in neutrophils neither in frequency nor median fluorescence intensity (MFI) in healthy controls (HC) (Additional file 1: Fig. A. S 2, *p* = 0.787 and *p* = 0.331 respectively). In addition, suPAR could not enhance ROS production in neutrophils without E.coli stimulation (*p* > 0.05, Additional file 1: Fig. A. S 3). Consistent with this point, there was no significant correlation between the spontaneous ROS production in neutrophils and serum suPAR levels (*p* > 0.05, Additional file 1; Fig. A. S 4) in patient with ACLF.

## Discussion

In this study, we investigated the role of suPAR in predicting short-term outcomes in patients with ACLF. Results showed that suPAR was an independent predictor for the short-term mortality of patients with ACLF. One of the underlying mechanisms might be suPAR enhanced neutrophil ROS production in ACLF under *E.coli* stimulation.

Bacterial infection is a main predisposing factor for the onset of ACLF [26], and the subsequent excessive inflammatory response is the driving factor for the occurrence of MOF. Our study showed the suPAR is associated with several infection-immunity-related indicators in patients with ACLF, suggesting that suPAR may play an important role in the pathogenesis of ACLF. We found a mild correlation between suPAR and bacterial or fungal infection, the latter reportedly causing the release of suPAR from the monocyte membrane [27]. Because this association was mild, it implies that there were other factors driving the production of circulating suPAR.

Recently, patients with ACLF were found to have higher suPAR levels than healthy controls. Because the ACLF patients displayed no signs of bacterial infection, it was assumed that either liver-derived factors induced uPAR cleavage from various immune cells, or that uPAR was shed from damaged or activated hepatocytes [28]. Since uPAR was not detected on damaged hepatocytes [28], the latter assumption may be excluded.

Excessive systemic inflammation is a notable feature of ACLF. suPAR, which acts as a chemokine, has been shown to play an important role in the immune system [29]. In addition, our study discovered a strong positive relationship between suPAR and two chemokines, MIP1beta and IL-8. This indicates that hepatic inflammation may be linked to suPAR release. However, no correlation was found between suPAR and other cytokines, such as IL-6 and IL-1. IL-6 and IL-8 have both been associated with short-term mortality in ACLF patients [30]. Since IL-1 and IL-6 were potent inducers of the acute phase response [30], this phenomenon may imply that suPAR was not directly involved in the onset of ACLF but may correlate with the later accumulation of immune cells in the liver. In addition, our study found suPAR increased ROS production in neutrophils in patients with ACLF.

(suPAR) is newly emerged a circulating factor that could predict the development and progression of chronic kidney disease CKD [31], such as focal segmental glomerulosclerosis (FSGS) [16, 32], which is characterized by proteinuria and associated with renal failure and kidney transplantation [33]. The underlying mechanism has been demonstrated that the circulating suPAR activates α_v_β_3_ integrin on podocyte membrane and leads to podocyte foot process effacement and damage glomerular barrier function [16]. APOL1 risk variants could synergize this process [34]. Such role of suPAR in kidney disease might explain our finding that the differences in plasma suPAR levels, when compared ACLF with or without complications, were most pronounced with hepatorenal syndrome (HRS).

In ACLF, the excessive immune response is due to overactivation of the innate immune system but not the adaptive immune system. Neutrophils and monocytes increased, but lymphocytes decreased, and the neutrophil-to-lymphocyte ratio (NLR) was positively associated with 90-day mortality [30]. Our study found that suPAR was positively associated with the percentage of neutrophil, indicating that suPAR is mainly derived from circulating neutrophils. In addition, the inverse relationship of suPAR to the percentage of lymphocytes and HBcAbs suggested that suPAR might be associated with the weak adaptive immunity of patients with ACLF. However, associations of suPAR with the percentage of those white cells were weak, indicating other factors such as the activities of immune cells might also influence the release of suPAR.

Patients with severe liver fibrosis have been shown to exhibit higher serum suPAR levels compared with patients with mild fibrosis [35, 36]. However, we found no difference in plasma suPAR levels between ACLF patients with or without cirrhosis. This may be because the immune response in patients with ACLF is so strong that the impact of fibrosis on suPAR was masked. K-M survival analysis revealed the interesting phenomenon that suPAR predicted the short-term outcome of patients with ACLF but without cirrhosis better than those with both ACLF and cirrhosis. This may be because the immune cells are in prolonged contact with suPAR, and these cells become insensitive to suPAR stimulation in patients with cirrhosis.

The association between ROS and suPAR has been barely studied. Kim et al. discovered a marked elevation in ROS levels in immortalized mouse podocytes, after treatment with recombinant suPAR for 24 h [37]. Our data suggest that suPAR could also enhance oxidative stress in neutrophils under *E.coli* stimulation in ACLF. The underlying mechanisms might relate to increased assembly of active cell surface NADPH oxidase 2 complexes [37]. Beside, our previous study showed neutrophils from HBV-related ACLF had much more ROS production under *E.coli* stimulation than HC and CHB, implying there was an immune disorder in neutrophils of ACLF [25]. Such immune disorder might explain why suPAR only impacted ROS production in neutrophils from ACLF but not those from HC or CHB.

The production of ROS (reactive oxygen species) was a key factor in the recruitment of activated neutrophils and monocytes to the liver by activated Kupffer cells in liver injury [18]. In addition to recruiting immune cells, ROS itself is also a toxic mediator, through which inflammatory cells can kill targets, such as bacteria, hepatocytes and other organ cells [18]. During the inflammatory response, ROS-induced cell killing mechanisms include the promotion of mitochondrial dysfunction [18]. Through intracellular oxidative stress, cell damage increases and cell contents are released, which further expands the scope of inflammatory damage [18]. ROS also promotes the secretion of cytokines, which in turn leads to an increase in ROS production, leading to a vicious circle and promoting the pathogenesis of liver disease [19]. In addition, resting ROS ≥ 12% in neutrophils predicts the 90-day mortality of patients with liver cirrhosis with high sensitivity and specificity [38]. All these indicate an essential role of ROS in ACLF pathogenesis, which in turn suggested elevated serum suPAR levels played a role of promoting disease progress in HBV related-ACLF.

Since suPAR is easy and fast to measure compared with the complicated scoring system, it has the potential to replace the complicated scoring systems in the busy emergency department or is incorporated to those scores to improve the predicting ability.

There are some limitations in our study. First, as was mentioned above, plasma suPAR levels were significantly higher in patients with renal failure compared to those without renal failure. In addition to the possibility that the kidneys secreted extra suPAR, it is also possible that renal failure made it difficult to remove suPAR from the circulation. Further studies are needed to determine why suPAR was elevated in patients with renal failure and whether the predictive ability of suPAR was influenced by those patients. Second, the treatments were not uniform. Due to variation between physician practices, doses and treatment strategies may differ. For example, the timing and dose of vasopressors administered might be different during circulatory failure in different patients treated by different doctors. Finally, we did not deeply explore the underlying mechanism of how suPAR enhanced neutrophil ROS production in patients with ACLF. Further study should focus on this point.

## Conclusions

suPAR was a useful biomarker predicting short-term outcomes in patients with ACLF independent of MELD and SOFA scores. One of the potential mechanisms might be suPAR enhanced neutrophil ROS production under *E.coli* stimulation in patients with HBV-related ACLF, indicating suPAR might play a key role in the pathogenesis of HBV-related ACLF.

## Supplementary Information

**Additional file 1.** **Table A. 1.** Risk factors for short-term mortality by univariate Cox regression analysis. **Figure A. 1.** Comparison of plasma suPAR concentrations in different disease groups. No statistical significance was found between each pair of groups. **Figure A. 2.** The effect of suPAR on ROS production in neutrophils in HC and CHB under E.coli stimulation. Whole blood from healthy controls (n = 13) and patients with CHB (n = 12) was stimulated with *E. coli* in the presence of suPAR (50 ng/ml) or PBS for 30 minutes in vitro. ROS levels in neutrophils were determined. Statistical analyses were performed using the Wilcoxon signed-rank test. *p < 0.05; ns, not statistically significant. **Figure A. 3.** The effect of suPAR on ROS production in neutrophils in ACLF without *E. coli* stimulation. Whole blood from patients with ACLF (n = 10) was incubated with suPAR (50 ng/ml) or PBS for 30 minutes in vitro without *E. coli* stimulation. ROS levels in neutrophils were determined. Statistical analyses were performed using the Wilcoxon signed-rank test. ns, not statistically significant. **Figure A. 4.** The correlation of serum suPAR and spontaneous ROS production in neutrophils from ACLF. The spontaneous ROS production in neutrophils from patients with ACLF (n = 11) was directly determined without E.coli stimulation. Statistical analyses were performed using the using spearman correlation. **Supplementary methods:** The limitations of cytokine measurements. **Supplementary methods:** Combining MELD or SOFA with suPAR.

## Data Availability

All data generated or analysed during this study are included in this published article and its supplementary information files.

## Abbreviations

ACLF: Acute-on-chronic liver failure
HBV-related: ACLF hepatitis B virus-related ACLF
CHB: Chronic hepatitis B
HC: Healthy control
suPAR: Soluble urokinase plasminogen activator receptor
ACLF-MOF: ACLF complicated with multi-organ failure
CTP: Child—Turcotte-Pugh
SOFA: Sequential organ failure assessment
CRP: C-reactive protein
HE: Hepatic encephalopathy
INR: International normalized ratio
MELD: Model for End-stage Liver Disease
SIRS: Systemic inflammatory response syndrome
UGIB: Upper gastrointestinal bleeding
WBC: White blood cell count
PCT: Procalcitonin
MIP1beta: Macrophage inflammatory protein 1-beta,
MAP: Mean arterial pressure
hsTnI: Hypersensitive troponin I
GFR: Glomerular filtration rate
T3: Total triiodothyronine
FT3: Free triiodothyronine
MFI: Median fluorescence intensity
