# Supplementary material for: Soluble urokinase plasminogen activator receptor is associated with short-term mortality and enhanced reactive oxygen species production in acute-on-chronic liver failure
Source: BMC Gastroenterol. 2021 Nov 17;21:429. doi: 10.1186/s12876-021-02006-x (PMC8597314; doi:10.1186/s12876-021-02006-x)
Supplement: Supplementary file 1 — Additional file 1. Table A. 1. Risk factors for short-term mortality by univariate Cox regression analysis. Figure A. 1. Comparison of plasma suPAR concentrations in different disease groups. No statistical significance was found between each pair of groups. Figure A. 2. The effect of suPAR on ROS production in neutrophils in HC and CHB under E.coli stimulation. Whole blood from healthy controls (n = 13) and patients with CHB (n = 12) was stimulated with E. coli in the presence of suPAR (50 ng/ml) or PBS for 30 minutes in vitro. ROS levels in neutrophils were determined. Statistical analyses were performed using the Wilcoxon signed-rank test. *p < 0.05; ns, not statistically significant. Figure A. 3. The effect of suPAR on ROS production in neutrophils in ACLF without E. coli stimulation. Whole blood from patients with ACLF (n = 10) was incubated with suPAR (50 ng/ml) or PBS for 30 minutes in vitro without E. coli stimulation. ROS levels in neutrophils were determined. Statistical analyses were performed using the Wilcoxon signed-rank test. ns, not statistically significant. Figure A. 4. The correlation of serum suPAR and spontaneous ROS production in neutrophils from ACLF. The spontaneous ROS production in neutrophils from patients with ACLF (n = 11) was directly determined without E.coli stimulation. Statistical analyses were performed using the using spearman correlation. Supplementary methods: The limitations of cytokine measurements. Supplementary methods: Combining MELD or SOFA with suPAR. [file 12876_2021_2006_MOESM1_ESM.docx]

**Appendices**

**Table A. 1. Risk factors for short-term mortality by univariate Cox regression analysis**

| Variable | 90-day mortality | |  |  | 30-day mortality | |
| --- | --- | --- | --- | --- | --- | --- |
|  | *p* - value | HR (95% CI) |  |  | *p* - value | HR (95% CI) |
| Age | <0.001 | 1.03 (1.01-1.05) |  |  | 0.003 | 1.03 (1.01-1.04) |
| Gender | 0.064 |  |  |  | 0.224 |  |
| Ascites | 0.118 |  |  |  | 0.198 |  |
| Cirrhosis | 0.018 | 1.73 (1.10-2.74) |  |  | 0.085 |  |
| UGIB | <0.001 | 3.01 (1.76-5.14) |  |  | 0.001 | 2.91 (1.58-5.31) |
| HRS | 0.006 | 2.79 (1.35-5.80) |  |  | 0.009 | 2.87(1.30-6.32) |
| HE | <0.001 | 7.02 (4.26-11.5) |  |  | <0.001 | 7.73 (4.51-13.2) |
| SBP | 0.509 |  |  |  | 0.163 |  |
| Infection | 0.054 |  |  |  | 0.012 | 2.15 (1.18-3.90) |
| Sepsis | <0.001 | 15.7 (4.77-51.6) |  |  | <0.001 | 16.1 (4.92-53.2) |
| suPAR | <0.001 | 1.09 (1.06-1.10) |  |  | <0.001 | 1.08 (1.06-1.10) |
| WBC | <0.001 | 1.10 (1.06-1.15) |  |  | <0.001 | 1.12 (1.07-1.17) |
| Platelet | 0.766 |  |  |  | 0.719 |  |
| ALT | 0.169 |  |  |  | 0.121 |  |
| Albumin | 0.397 |  |  |  | 0.75 |  |
| Sodium | <0.001 | 0.92 (0.88-0.95) |  |  | 0.008 | 0.95 (0.92-0.99) |
| Bilirubin | <0.001 | 1.08 (1.05-1.11) |  |  | <0.001 | 1.08 (1.05-1.11) |
| INR | <0.001 | 1.65 (1.44-1.90) |  |  | <0.001 | 1.71 (1.48-1.97) |
| Creatinine | 0.002 | 1.55 (1.18-2.03) |  |  | 0.003 | 1.57 (1.17-2.13) |
| CTP | <0.001 | 1.71 (1.38-2.12) |  |  | <0.001 | 1.91 (1.49-2.44) |
| MELD | <0.001 | 1.14 (1.10-1.17) |  |  | <0.001 | 1.14 (1.10-1.18) |
| SOFA | <0.001 | 1.76 (1.51-2.06) |  |  | <0.001 | 1.88 (1.59-2.23) |

UGIB upper gastrointestinal bleeding, HRS hepatorenal syndrome, HE hepatic encephalopathy, SBP spontaneous bacterial peritonitis, WBC white blood cell count., INR international normalized ratio, CTP Child—Turcotte-Pugh, MELD Model for End-stage Liver Disease, SOFA sequential organ failure assessment.





**Figure A. 1 Comparison of plasma suPAR concentrations in different disease groups.** No statistical significance was found between each pair of groups.





**Figure A. 2 The effect of suPAR on ROS production in neutrophils in HC and CHB under *E.coli* stimulation.**

Whole blood from healthy controls (n=13) and patients with CHB (n=12) was stimulated with *E. coli* in the presence of suPAR (50ng/ml) or PBS for 30 minutes in vitro. ROS levels in neutrophils were determined. Statistical analyses were performed using the Wilcoxon signed-rank test. **p*<0.05; ns, not statistically significant.





**Figure A. 3 The effect of suPAR on ROS production in neutrophils in ACLF without *E.coli* stimulation.**

Whole blood from patients with ACLF (n=10) was incubated with suPAR (50ng/ml) or PBS for 30 minutes in vitro without *E.coli* stimulation. ROS levels in neutrophils were determined. Statistical analyses were performed using the Wilcoxon signed-rank test. ns, not statistically significant.





**Figure A. 4 The correlation of serum suPAR and spontaneous ROS production in neutrophils from ACLF.** The spontaneous ROS production in neutrophils from patients with ACLF (n=11) was directly determined without *E.coli* stimulation. Statistical analyses were performed using the using spearman correlation.

**Supplementary methods**

**The limitations of cytokine measurements.**

suPAR 1.0ng/mL, IL-1β, 17.3pg/mL; IL-1rα,15pg/mL; IL-2, 16.5pg/mL; IL-4, 11pg/mL; IL-5, 27pg/mL; IL-6, 24.8pg/mL; IL-7, 11pg/mL; IL-8, 14.5pg/mL; IL-9: 21pg/mL; IL-10: 33.3pg/mL; IL-12: 15.5pg/mL; IL-13: 13.5pg/mL; IL-5; 57.3pg/mL; IL-17: 24.8pg/mL; eotaxin, 17pg/mL; FGF basic, 14pg/mL; G-CSF, 36.3pg/mL; GM-CSF, 16pg/mL; IFN-γ, 15.5pg/mL; IP-10, 14.5pg/mL; MCP-1, 18.5pg/mL; MIP-1α, 8.8pg/mL; PDGF-bb, 32.5pg/mL; MIP-1β, 31.5pg/mL; RANTES, 15.8pg/mL; TNF-α, 12.5pg/mL; VEGF, 109.3pg/mL.

**Combining MELD or SOFA with suPAR**

Variables in the Equation—90-day follow up

|  | | B | SE | Wald | df | Sig. | Exp(B) | 95.0% CI for Exp(B) | |
| --- | --- | --- | --- | --- | --- | --- | --- | --- | --- |
|  |  |  |  |  |  |  |  | Lower | Upper |
| Step 2 | suPAR | .060 | .012 | 25.047 | 1 | .000 | 1.061 | 1.037 | 1.086 |
|  | MELD | .068 | .016 | 18.297 | 1 | .000 | 1.071 | 1.038 | 1.104 |

The predicting equation of combining of MELD with suPAR:

MELD-suPAR90=0.060*suPAR+0.68*MELD

Variables in the Equation—90-day follow up

|  | | B | SE | Wald | df | Sig. | Exp(B) | 95.0% CI for Exp(B) | |
| --- | --- | --- | --- | --- | --- | --- | --- | --- | --- |
|  |  |  |  |  |  |  |  | Lower | Upper |
| Step 1 | suPAR | .064 | .011 | 36.598 | 1 | .000 | 1.066 | 1.044 | 1.089 |
|  | SOFA | .259 | .050 | 26.972 | 1 | .000 | 1.296 | 1.175 | 1.429 |

The predicting equation of combining of SOFA with suPAR:

SOFA-suPAR90=0.064*suPAR+0.259*SOFA

Variables in the Equation—30-day follow up

|  | | B | SE | Wald | df | Sig. | Exp(B) | 95.0% CI for Exp(B) | |
| --- | --- | --- | --- | --- | --- | --- | --- | --- | --- |
|  |  |  |  |  |  |  |  | Lower | Upper |
| Step 2 | suPAR | .055 | .013 | 17.141 | 1 | .000 | 1.057 | 1.030 | 1.085 |
|  | MELD | .069 | .018 | 14.689 | 1 | .000 | 1.071 | 1.034 | 1.110 |

The predicting equation of combining of MELD with suPAR:

MELD-suPAR30=0.055*suPAR+0.69*MELD

Variables in the Equation—30-day follow up

|  | | B | SE | Wald | df | Sig. | Exp(B) | 95.0% CI for Exp(B) | |
| --- | --- | --- | --- | --- | --- | --- | --- | --- | --- |
|  |  |  |  |  |  |  |  | Lower | Upper |
| Step 2 | suPAR | .059 | .012 | 24.523 | 1 | .000 | 1.061 | 1.036 | 1.086 |
|  | SOFA | .279 | .057 | 24.256 | 1 | .000 | 1.322 | 1.183 | 1.477 |

The predicting equation of combining of SOFA with suPAR:

SOFA-suPAR30=0.059*suPAR+0.279*SOFA
